# Characterization of Curcumin/Cyclodextrin Polymer Inclusion Complex and Investigation on Its Antioxidant and Antiproliferative Activities

**DOI:** 10.3390/molecules23051179

**Published:** 2018-05-15

**Authors:** Jianping Chen, Xiaoming Qin, Saiyi Zhong, Suhua Chen, Weiming Su, Ying Liu

**Affiliations:** 1 College of Food Science and Technology, Guangdong Ocean University, Guangdong Provincial Modern Agricultural Science and Technology Innovation Center for Subtropical Fruit and Vegetable Processing, Zhanjiang 524088, China; cjp516555989@126.com (J.C.); xiaoming0502@21cn.com (X.Q.); zsylxc@126.com (S.Z.); cshh1111@126.com (S.C); hdsuwm@163.com (W.S.); 2 Guangdong Province Key Laboratory for Green Processing of Natural Products and Product Safety, Guangzhou 510640, China; 3 Faculty of Agricultural Science, Guangdong Ocean University, Zhanjiang 524088, China

**Keywords:** curcumin, β-cyclodextrin polymer, inclusion complex, antioxidant activity, anticancer activity

## Abstract

The aims of this study were to characterize the curcumin/cyclodextrin polymer inclusion complex using X-ray diffractometry (XRD), Fourier transform infrared spectroscopy (FTIR), differential scanning calorimetry (DSC), and UV–vis spectroscopy, and to determine the antioxidant activity of this complex by methods of scavenging 2,2-azinobis-3-ethylbenzothiazoline-6-sulfonic acid (ABTS) radicals assays and 1,1-diphenyl-2-picrylhydrazyl (DPPH) radicals assays. The inhibitory effect of inclusion complex on A375 cells was also investigated by CCK-8 assay, Annexin-V/PI staining assay, and caspase activity assay. The results showed that the complex exhibited different physicochemical characteristics from that of free curcumin. Moreover, the inclusion complex exhibited novel antioxidant activity by scavenging the ABTS and DPPH free radicals and displayed higher antiproliferative activity on A375 cells. Further investigation revealed that inclusion complex could induce A375 cell apoptosis. These findings suggest that inclusion complex could be developed as a novel natural antioxidant with potential applications in cancer chemoprevention.

## 1. Introduction

Curcumin (C_21_H_20_O_6_), a hydrophobic yellow-orange polyphenol derived from the rhizome of the herb Curcuma longa, has been widely used in food as a stabilizer in jellies or as a natural colorant in cheeses and in traditional medicine as an ingredient [1,2]. Additionally, curcumin has been shown to inhibit the growth and proliferation of variety of tumor cells [3,4]. However, the disadvantage of poor bioavailability and rapid metabolism of curcumin has restricted its application. Various techniques such as liposomes, nanocapsules, nanoparticles, and various derivatives of cyclodextrins have been tried to enhance curcumin delivery [5]. Among these methods, inclusion complexation with cyclodextrins and their derivatives are the most useful method to enhance the aqueous solubility of poorly water-soluble bioproducts [6].

Cyclodextrins (CDs) are cyclic oligomers of glucose that can form water-soluble inclusion complexes with small molecules or fragments of large compounds [7]. Nowadays, many studies on enhancement of solubility of curcumin with cyclodextrins have been reported. Although there are many studies of curcumin with CDs to enhance the guest’s solubility [2,8], the limited solubility of host CDs restrains the application of curcumin in water. Therefore, finding a high water-soluble host is a key to extensive applications of curcumin in aqueous phase. β-cyclodextrin polymer has been obtained by reaction of the parent β-cyclodextrin with a cross-linking agent, epichlorohydrin [9]. Especially, β-cyclodextrin polymer as a highly water-soluble polymer is well known to selectively form inclusion complexes. Zhang et al. have reported that β-cyclodextrin polymer was used as a host molecule to prepare hypericin/β-cyclodextrin polymer inclusion complex to improve its aqueous solubility [10].

Therefore, in this article, the curcumin/β-cyclodextrin polymer inclusion complex was prepared according to the previous method and evaluated the formation of the complexes using the FTIR, X-ray diffraction, DSC, and UV. Additionally, the antioxidant and antiproliferative activities of the formed complexes were further investigated.

## 2. Results and Discussion

### 2.1. Physicochemical Characterization of Curcumin/Cyclodextrin Polymer Inclusion Complex

#### 2.1.1. XRD Analysis

**Figure 1:**
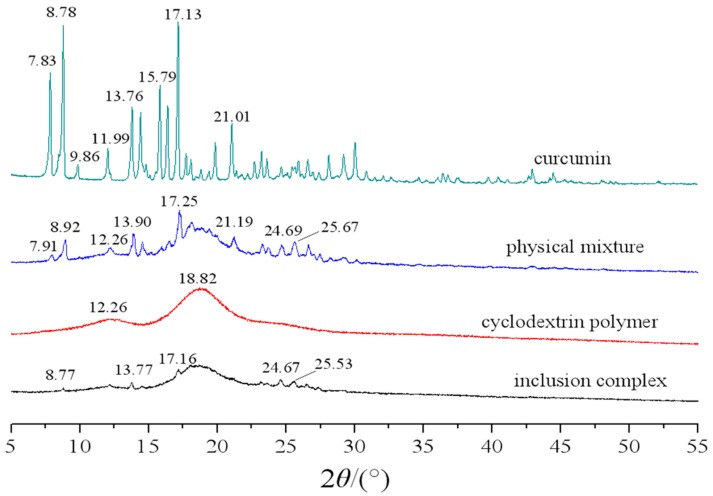
Powder X-ray diffraction patterns of curcumin, curcumin and cyclodextrin polymer physical mixture, cyclodextrin polymer, and curcumin/cyclodextrin polymer inclusion complex.

The XRD spectra of curcumin, cyclodextrin polymer, their physical mixture and inclusion complex are shown in Figure 1. As shown in Figure 1, some sharp peaks at the diffraction angle of 2θ 7.83, 8.78, 9.86 11.99, 13.76, 15.79, 17.13, and 21.01 were present in the X-ray diffractogram of curcumin powder, suggesting that the powder exists in a crystalline form. The XRD pattern of cyclodextrin polymer showed two broad peaks in the ranges of 10–15° and 15–20° (2θ), confirming its amorphous characteristic in nature. The X-ray diffractogram of the physical mixture showed approximate superimposition of the individual patterns of both cyclodextrin polymer and curcumin. In the case of inclusion complex, the spectrum exhibits a new diffraction peak. Some sharp peaks originally found at the diffraction angles of 2θ 7.83, 8.78, 9.86 11.99, 13.76, 15.79, 17.13, and 21.01 in the curcumin samples disappeared or weakened, suggesting the formation of the inclusion complex.

#### 2.1.2. FTIR Analysis

**Figure 2:**
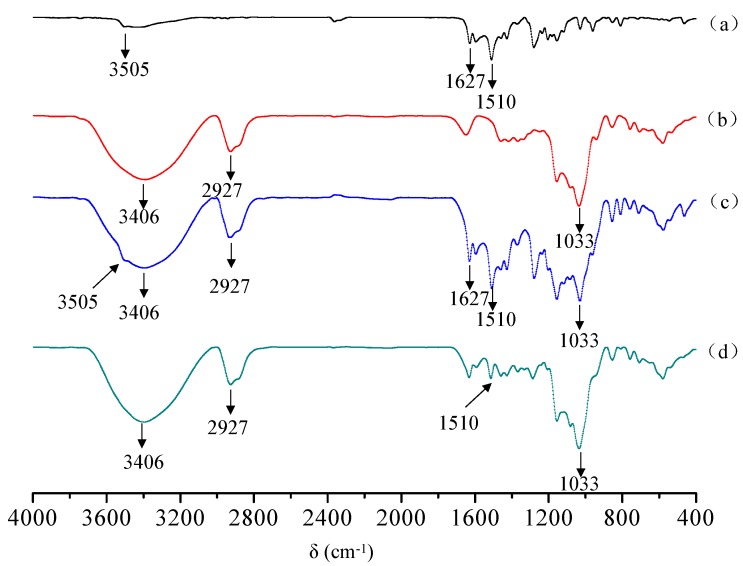
FTIR spectra of curcumin (**a**); cyclodextrin polymer (**b**); curcumin and cyclodextrin polymer physical mixture (**c**); curcumin/cyclodextrin polymer inclusion complex (**d**).

FTIR spectroscopy was used to ascertain the formation of the curcumin/cyclodextrin polymer inclusion complex. Figure 2 shows the FTIR spectra of curcumin, cyclodextrin polymer, their physical mixture and inclusion complex. The typical IR spectrum of curcumin was presented in Figure 2a, which was in good agreement with literature [11]. The FTIR spectrum of curcumin exhibits an absorption band at 3505 cm^−1^ indicate the presence of the phenolic O-H stretching vibration. Additionally, stretching vibrations of benzene ring of curcumin at 1627 cm^−1^ and C-O and C-C vibrations of curcumin at 1510 cm^−1^ was exhibited in Figure 2a. The typical IR spectrum of cyclodextrin polymer was presented in Figure 2b, which was in good agreement with literature [12]. In Figure 2b, the bands observed at 3406 cm^−1^ and 2927 cm^−1^ are assigned to -OH stretching vibration and -CH2 anisomerous stretching vibration, respectively. The characteristic band observed at 1033 cm^−1^ is assigned to stretching vibration of C-O-C. All above absorption peaks can be found in physical mixtures of curcumin and cyclodextrin polymer (Figure 2c). However, all absorption peaks of cyclodextrin polymer can be found, but all characteristic peaks of curcumin almost disappear. Only a faint C-O and C-C vibrations at 1511 cm^−1^ was observed (Figure 2d), which provides substantial evidence of the formation of curcumin/cyclodextrin polymer inclusion complex.

#### 2.1.3. DSC Analysis

**Figure 3:**
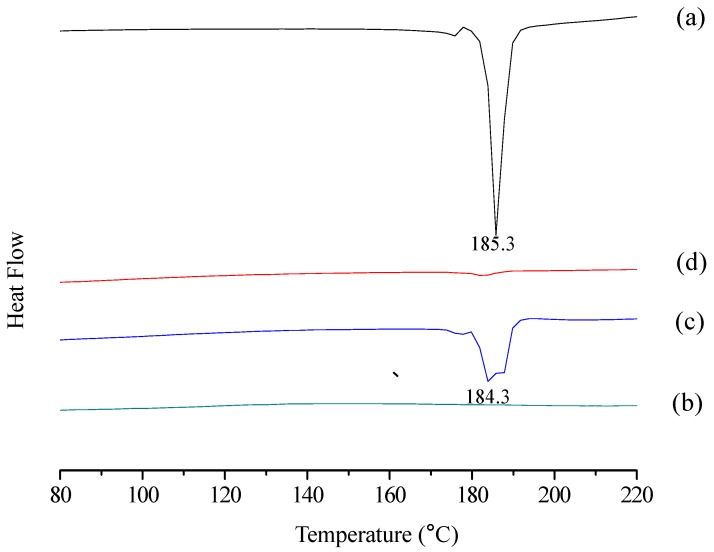
DSC thermograms of curcumin (**a**); cyclodextrin polymer (**b**); curcumin and cyclodextrin polymer physical mixture (**c**); curcumin/cyclodextrin polymer inclusion complex (**d**).

DSC curves of curcumin, cyclodextrin polymer, their physical mixture and inclusion complex were shown in Figure 3. Curcumin showed a sharp endothermic peak at 185.3 °C, corresponding to the melting point (Figure 3a). In contrast, the thermogram of cyclodextrin polymer did not present any peak in the region of 80–220 °C (Figure 3b). The thermal profile of their physical mixture was apparently a combination of characteristics of curcumin and cyclodextrin polymer (Figure 3c). However, the DSC curve of their inclusion complex mainly showed features of cyclodextrin polymer, while the DSC characteristics of curcumin disappeared (Figure 3d). These data should confirm the formation of the inclusion complex.

#### 2.1.4. UV Spectra Analysis

**Figure 4:**
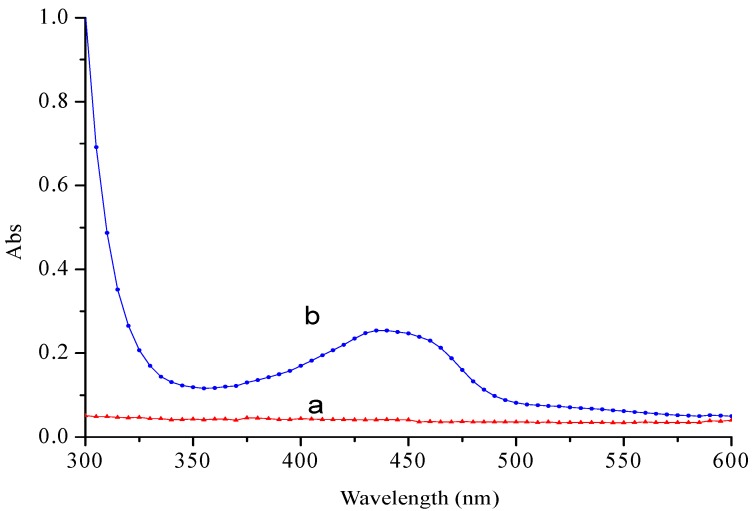
UV spectra of cyclodextrin polymer (**a**) and curcumin/cyclodextrin polymer inclusion complexes (**b**) at a wavelength of 300–600 nm at room temperature.

UV spectra of curcumin/cyclodextrin polymer inclusion complexes and cyclodextrin polymer were shown in Figure 4. In Figure 4a, no absorption was observed for cyclodextrin polymer in the range of 300–600 nm, while the typical absorption peaks in the inclusion complex were observed in the range of 300–600 nm as shown in Figure 4b, which attributed to the absorption peak of curcumin. These results indicated that the inclusion complex was formed.

### 2.2. Phase Solubility of Curcumin/Cyclodextrin Polymer Inclusion Complex

**Figure 5:**
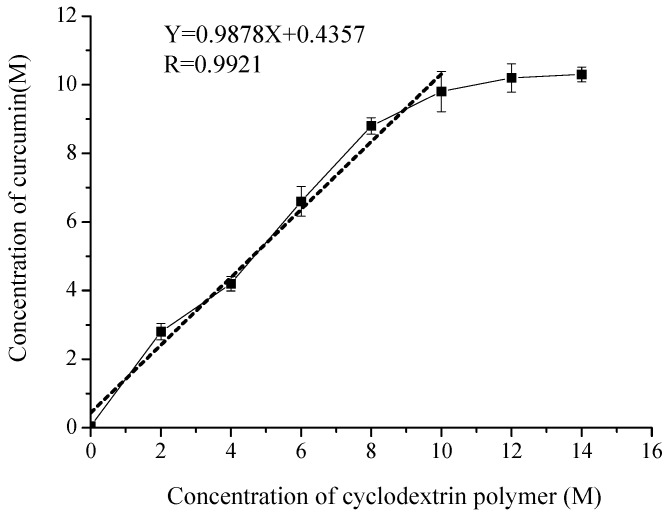
Phase solubility profile of curcumin/cyclodextrin polymer inclusion complex.

Phase solubility analysis is very useful for investigating inclusion complexation of poorly soluble compounds with cyclodextrin polymer, because it gives not only the stoichiometry of curcumin/cyclodextrin polymer inclusion complex but also the stability constant of the complexes by analyzing the solubility curve [13]. The phase solubility diagrams for curcumin/cyclodextrin polymer inclusion complex is shown in Figure 5, which displayed an AL-type according to Higuchi and Connors [14]. According to Higuchi and Connors’ theory, the 1:1 stoichiometry of the inclusion complex was achieved from the initial ascending part of the curve, a nearly straight line with the slope of 0.9878. The regression equation was

Y = 0.9878X + 0.4357, R = 0.9921

where Y is the concentration (M) of curcumin, X is the concentration (M) of cyclodextrin polymer. The apparent stability constant K_1:1_ was obtained to be 198 M^−1^ according to Equation (1), which indicated that a sufficient interaction between curcumin and cyclodextrin polymer occurs.

### 2.3. In-Vitro Antioxidant Activity of Curcumin/Cyclodextrin Polymer Inclusion Complex

**Figure 6:**
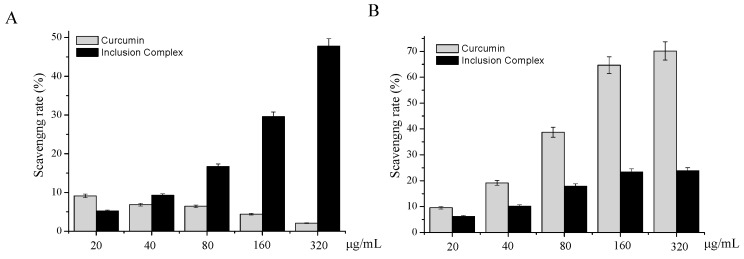
ABTS radical scavenging rate (**A**) and DPPH radical scavenging rate (**B**) of curcumin/β-cyclodextrin polymer inclusion complex or curcumin with different concentrations. Values expressed are means ± SD of triplicates.

The total antioxidant capacity of inclusion complex was evaluated by ABTS•+ scavenging methods. The relatively long-lived ABTS•+, generated by the direct oxidation of ABTS with manganese dioxide, is decolorized during the reaction with hydrogen-donating antioxidants [15]. As shown in Figure 6A, the ABTS radical scavenging rate of curcumin was extremely low. However, comparing with curcumin, the ABTS radical scavenging rate of inclusion complex was significantly increased. With the concentration of inclusion complex increased, the ABTS radical scavenging rate of inclusion complex was increased from 5.28 ± 0.21% (20 μg/mL) to 47.79 ± 0.91% (320 μg/mL). Our results indicated that inclusion complex could effectively scavenge the ABTS free radical in a dose-dependent manner.

The DPPH method is used worldwide to quantify free radical scavenging activity. The method is based on the loss of color when the odd electron of the nitrogen atom in the DPPH radical is reduced by receiving a hydrogen atom from an antioxidant compound [16]. The antioxidant capacity of bioactive compounds can be modified in the presence of cyclodextrins. Therefore, the antioxidant activity of inclusion complex can be determined by monitoring the drop in absorbance by DPPH radical at 517 nm. The DPPH radical scavenging activities of curcumin and inclusion complex are shown in Figure 6B. With the concentration of inclusion complex increased, the DPPH radical scavenging rate of inclusion complex was increased from 6.18 ± 0.31% (20 μg/mL) to 23.85 ± 1.19% (320 μg/mL). It showed that the scavenging activities of inclusion complex on DPPH radical were dose dependent. However, the scavenging activity of inclusion complex is lower than the curcumin.

### 2.4. In-Vitro Anticancer Activity of Curcumin/Cyclodextrin Polymer Inclusion Complex

#### 2.4.1. CCK-8 Assay

The CCK-8 assay is a commonly used method to detect cell survival. To evaluate the cytotoxicity of inclusion complex, the A375, A549, HeLa, and MCF-7 cell lines were chosen to investigate the in vitro cytotoxicity of inclusion complex. The A375, A549, HeLa, and MCF-7 cell lines were exposed to various concentrations of inclusion complex (40, 80, 160, 320, 640 μg/mL) for 72 h followed by a CCK-8 assay. The results are shown in Table 1. As shown in Table 1, inclusion complex exhibited stronger inhibitory effects on A375 cells than other cancer cells, as shown by the IC_50_ values of 476.4 μg/mL for A375 cells, 517.2 μg/mL for A549, 545.7 μg/mL for Hela, 692.8 μg/mL for MCF-7. The A375 cells were selected to examine the antiproliferative activity of inclusion complex.

**Figure 7:**
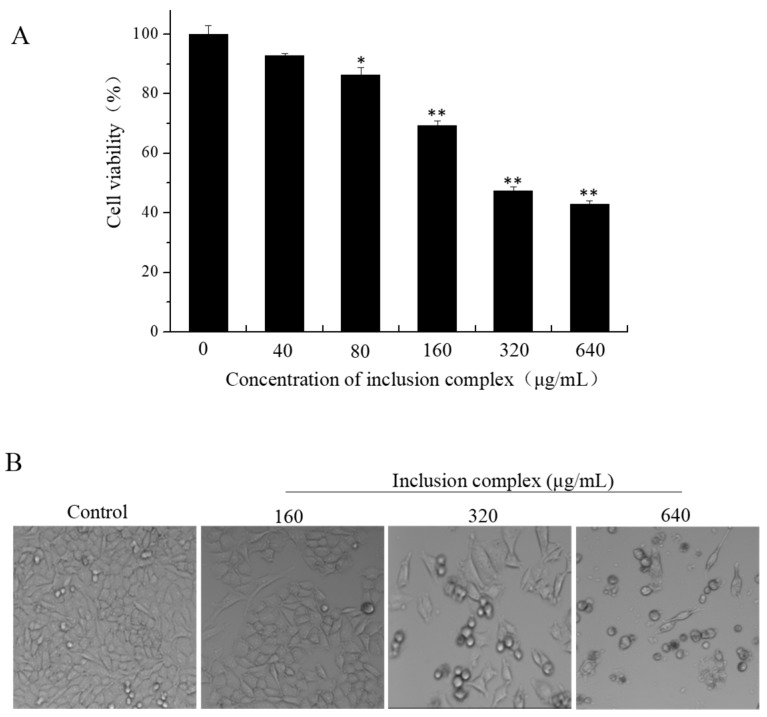
Effect of inclusion complex treatment for 72 h on cell viability of A375 cells. (**A**) Dose-dependent cytotoxic effects of inclusion on A375 cells. The cells were treated with different concentrations of inclusion complex and incubated for 72 h. Cell viability was determined by the CCK-8 assay. All data were obtained from three independent experiments are presented as the means ± SD. *p* < 0.05 (*) or *p* < 0.01 (**) vs. control group; (**B**) Morphological changes of A375 cells as examined by phase-contrast microscopy (magnification, 200×). The images shown here are representative of three independent experiments with similar results.

As shown in Figure 7A, the cell viability of A375 were in a dose dependent decrease with the concentration of inclusion complex ranging from 40 to 640 μg/mL. After the A375 cell lines were respectively exposed to inclusion complex for 72 h, the cell viability decreased from 92.7% ± 0.9% at 40 μg/mL to 43.0 ± 1.2% at 640 μg/mL. Our results demonstrated that inclusion complex exhibited cytotoxic effects on the A375 cell lines. The morphological changes on A375 cells were attenuated by different concentrations of inclusion complex, given in Figure 7B. With increasing concentration of inclusion complex, the number of cells decreased gradually and the cells’ morphology become round by comparison with control cells. This result was consistent with the detection of cell viability.

#### 2.4.2. Annexin V/PI Staining Assay

**Figure 8:**
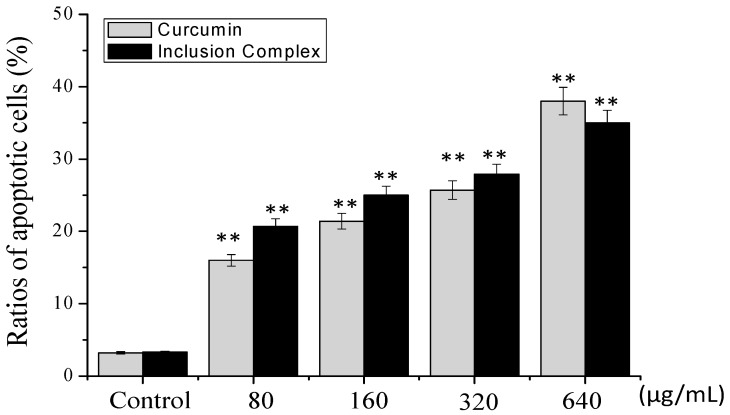
Flow cytometric analysis of A375 cells treated with inclusion complex by Annexin V/PI staining assay. *p* < 0.01 (**) vs. control group.

Translocation of phosphatidylserine (PS) from the inner cellular membrane to the outer leaflet in the early stage of apoptosis has been identified as an important characteristic of apoptosis [17]. In order to further confirm that the cell death induced by inclusion complex was apoptosis, Annexin V/PI double staining was used to detect the extroversion of phosphatidyl serine on the cell membrane and the PI-specific stained cell nuclei after rupture of the membrane, indicating apoptosis. The results are shown in Figure 8. As shown in Figure 8, treatment of curcumin increased the percentage of apoptotic cells from 2.9% (control) to 16.0% (80 μg/mL), 21.4% (160 μg/mL), and 25.7% (320 μg/mL), respectively. However, compared with the treatment of curcumin, the inclusion complex significantly increased the cell apoptosis from 16.0% to 20.7%, 21.4% to 25.0%, and 25.7% to 27.9%, respectively. Our results indicated that inclusion complex promotes apoptosis on A375 cells in a dose-dependent manner.

#### 2.4.3. Caspase Activity

**Figure 9:**
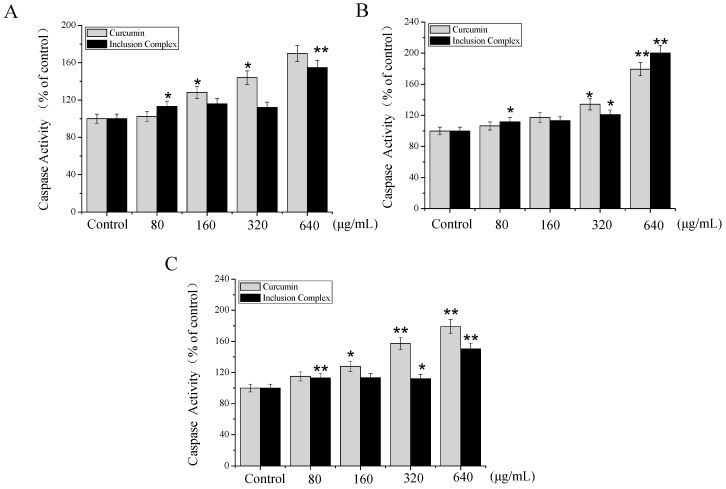
Effects of inclusion complex on caspase activities of caspase 3 (**A**), caspase 8 (**B**), and caspase 9 (**C**) in A375 cells. Caspase activities as measured by specific fluorescent substrates for caspase-3/8/9. Cells were pretreated with different concentrations of inclusion complex or curcumin for 72 h, respectively. Significant difference between treatment groups and control groups is indicated at *p* < 0.05 (*) or *p* < 0.01 (**) level.

According to the above experiments, inclusion complex induced the A375 cells apoptosis. However, the underlying mechanism was not clear. Many studies have shown that the two most important pathways were involved in inducing cell apoptosis including the death receptor-mediated extrinsic pathway and the mitochondria-mediated intrinsic pathway [18]. The caspase family members play a vital role in two central apoptotic pathways. Among them, caspase-8/9 acts as the initiator of the extrinsic and intrinsic pathways, respectively, whereas caspase-3 is regarded as the central executioner of apoptosis [19]. To further confirm whether caspases-3/8/9 were involved in apoptosis, the activation of caspase-3/8/9 was analyzed by fluorometric assay. As shown in Figure 9, treatment of inclusion complex increased the activation of caspase-3/8/9 to some extent. However, comparing with curcumin at 640 μg/mL, inclusion complex decreased the activation of caspase-3/9 and increased the activation of caspase 8. Our results indicated that inclusion complex induced apoptosis in A375 cells though extrinsic and intrinsic apoptotic pathways.

## 3. Materials and Methods

### 3.1. Materials

Curcumin, ABTS (2,2′-azinobis-3-ethylbenzothiazolin-6-sulfonic acid), and DPPH (1,1′-diphenyl-2-picryhydrazyl) were obtained from Sigma-Aldrich (Sigma-Aldrich, St. Louis, MO, USA). β-CD (chemical purity) was purchased from Shangdong Xinda Biotechnology Co., Ltd. (Zibo, China). Epichlorohydrin (EP) and ethylene glycol were purchased from Sinopharm Chemical Reagent Co., Ltd. (Shanghai, China). RPMI 1640 medium were purchased from Hyclone Company (South Logan, UT, USA). Fetal bovine serum (FBS) and the antibiotic mixture (penicillin-streptomycin) were purchased from Gibco Company (Grand Island, NY, USA). Cell Counting Kit-8 (CCK-8) was purchased from Dojindo Company (Kumamoto, Japan). Caspase 3 assay kit (ab39401), Caspase 8 assay kit (ab39700), and Caspase 9 assay kit (ab65608) were purchased from Abcam Company (Cambridge, UK). Annexin V/PI apoptosis kit were purchased from MultiScience Company (Hangzhou, China). Sodium hydroxide and other reagents were of analytical grade and were purchased from Nanjing Chemical Reagent Co. Ltd. (Nanjing, China). Double distilled and sterilized water was used to prepare all solutions.

### 3.2. Preparation of Cyclodextrin Polymer and Curcumin/Cyclodextrin Polymer Inclusion Complex

Cyclodextrin polymer was synthesized by cross-linking β-CD with EP under a strongly alkaline condition (33 wt % NaOH). The molar ratio of β-CD/EP was 1:7. The details of the synthesis and purification were shown as in the reference [9].

The preparation of the inclusion complex of curcumin with cyclodextrin polymer was shown as follow: briefly, 4 g cyclodextrin polymer and 1 g curcumin was mixed together by a spatula in the mortar, then dissolved in 50 mL of distilled water. The resultant mixture was treated by SB-3200 ultrasonic (Xinzhi Biotechnology, Ningbo, China) for 5 min, then sufficiently stirred for at least 48 h at room temperature. At the end of the reaction, the final solution was recovered by filtration to remove the residual curcumin. Then, the inclusion complex was obtained by pressure distillation, and it was dried in a vacuum oven at 60 °C for 48 h.

### 3.3. Preparation of Curcumin and Cyclodextrin Polymer Physical Mixture

Cyclodextrin polymer was pulverized in ceramic mortars. The calculated amounts of curcumin and cyclodextrin polymer with a mass ratio of 1:4 were mixed together by a spatula until a homogeneous mixture was obtained.

### 3.4. Physicochemical Characterization

#### 3.4.1. Fourier Transform Infrared Spectroscopy (FTIR)

The FTIR spectra of curcumin, cyclodextrin polymer, their physical mixture, and the inclusion complex were obtained using a Tensor 27 fourier transform infrared spectroscopy (Bruker, Karlsruhe, Germany) with 256 scans at a resolution of 4 cm^−2^. The scanning range was 4000 and 400 cm^−1^. The samples were ground with spectroscopic grade potassium bromide (KBr) powder and pellets were made to perform the measurements.

#### 3.4.2. X-ray Diffractometry (XRD)

The X-ray diffraction was recorded using D8 ADVANCE diffractometer (Bruker, Germany), operated at a voltage of 40 KV and a current of 40 mA. The samples were investigated in the 2θ angle range of 5–55° and the process parameters were set as: scan step size of 0.02°, scan step time of 17.7 s.

#### 3.4.3. Differential Scanning Calorimetry (DSC)

DSC curves of curcmin, cyclodextrin polymer, the physical mixture and the inclusion complex were analyzed by a STA449F3 simultaneous thermal analyzer (Netzsch, Bavaria, Germany). Each sample (2–5 mg) was heated in a sealed aluminum pan at a rate of 10 °C/min from 80 to 220 °C under a nitrogen flow of 30 mL/min. An empty sealed pan was used as the reference.

#### 3.4.4. UV Analysis

10 mg of cyclodextrin polymer and inclusion complex were diluted in 100 mL of water, respectively. They were scanned using an Evolution 300 UV–vis spectrophotometer (Thermo Scientific, Waltham, MA, USA) at a wavelength of 300–600 nm at room temperature.

### 3.5. Phase Solubility Study

Phase solubility analysis was carried out according to the method reported by Higuchi and Connors [14]. Briefly, an excess amount of curcumin was added into 10 mL of cyclodextrin polymer aqueous solutions with various concentrations (2–14 mol/L). Then all the samples were then shaken at (25 ± 2) °C for 48 h. After equilibrium was reached, the mixtures were withdrawn and filtered through 0.45 μm membrane filter and properly diluted. The concentration of curcumin in each filtrate was determined by a high performance liquid chromatography (HPLC). The apparent complexation constant (Kc) for complexation could be calculated from the phase solubility diagrams, according to the following equation (Equation (1)): *Kc* = slope/S_0_(1-slope).

Where S_0_ is the intrinsic solubility of curcumin in deionized water in the absence of cyclodextrin polymer and slope is the slope of the straight line.

### 3.6. HPLC Analysis

Curcumin (12.25 mg) was dissolved in methanol (100 mL). Then 0.2, 0.4, 0.6, 0.8, 1.6, 2.4, 3.2, and 5.0 mL solution were taken out and diluted to 10 mL with mobile phase, respectively. These samples were analyzed by a Waters HPLC system (Waters 2695N, Milford, MA, USA) with methanol −1% citric acid (70:30, *v/v*) as the mobile phase. The flow rate of mobile phase was 1 mL/min. 10 μL samples were separated on a Scienhome Kromasil C18 column (4.6 × 150 mm, 5 μm) and detected at 425 nm. According to the calibration curve: Y = 102211X − 256580, R = 0.9940, the concentration of curcumin was calibrated, and a good linearity was observed in the range of 2.45–49 μg/mL.

### 3.7. Antioxidant Activities of Curcumin/Cyclodextrin Polymer Inclusion Complex

#### 3.7.1. ABTS+ Free Radical Scavenging Assay

According to previous research methods by Miller et al. [20], 5 mM ABTS stock solution in PBS (pH 7.4) and the right amount of MnO_2_ were mixed until full reaction. Then the solution was filtrated by 0.2 μM PVDF syringe filter and diluted with 5 mM phosphate-buffered saline (PBS) buffer (pH 7.4) appropriately to obtain ABTS reaction solution (an absorbance of 0.70 at 734 nm recorded on a Varioskan Flash multimode reader). In the reaction system, 180 μL ABTS reaction solution and 20 μL samples were added to 96-well plates. After mixing for 5 min, the absorbance of mixture was measured at 734 nm using a Varioskan Flash multimode reader (Thermo Fisher Scientific, USA) with room temperature in the dark.

#### 3.7.2. Scavenging Activity of DPPH+ Free Radical

According to methods of previous study by Okada and Okada [21], a certain amount of DPPH was dissolved in methanol to obtain a stock solution of DPPH (6 mM), stored at −20 °C in the dark. The solution was diluted with methanol to a final concentration of 60 μM before using. In the reaction system, 180 μL DPPH reagent and 20 μL samples were added to 96-well plates, mixed, and shocked for 5 min. The absorbance was recorded at 515 nm by Varioskan Flash microplate reader (Thermo Fisher Scientific, USA) until the reading was stable. This experiment was carried out at room temperature in the dark.

### 3.8. Evaluation of Cytotoxic Activity of Curcumin/Cyclodextrin Polymer Inclusion Complex

#### 3.8.1. Cell Culture

A375 melanoma cell line, A549 lung carcinoma cell line, HeLa cervical carcinoma cell line and MCF-7 breast adenocarcinoma cell line were purchased from American Type Culture Collection (ATCC, Manassas, VA, USA). The cell lines were grown in RPMI-1640 medium supplemented with 10% fetal bovine serum, 100 units/mL penicillin, and 50 units/mL streptomycin at 37 °C in a humidified (5% CO_2_, 95% air) atmosphere.

#### 3.8.2. Cell Viability Assay

Cell viability assay was analyzed using Cell Counting Kit (CCK-8; Dojindo Laboratories, Kumamoto, Japan) assay in accordance with the manufacturer’s protocol. Briefly, cells were seeded into 96-well culture plates (1 × 10^4^ cells/well) and then cultured at 37 °C under saturated humidity incubator (5% CO_2_, 95% air) for 24 h. The cells were treated with inclusion complex at different concentrations for 72 h. After incubation, the medium in the 96-well plate was replaced with fresh medium, and 10 μL CCK-8 working fluid was added to each well. After a 4 h culture in the incubator, the optical density (OD) value (absorbance) was measured at 450 nm by a microplate spectrophotometer (Multiskan, MK3). All experiments were performed in quadruple on three separate occasions.

#### 3.8.3. Annexin-V/PI Staining Assay

We used an Annexin V/PI apoptosis kit from MultiScience Company (Hangzhou, China) to detect cell apoptosis induced by inclusion complex as previously described [22]. In brief, following the kit instructions, A375 cells were treated with inclusion complex or curcumin at different concentrations for 72 h. After reaching the specified time, the cells were trypsinized, collected by centrifugation at 1000 rpm for 5 min and washed twice with PBS. The cells were resuspended in 500 μL binding buffer and stained with 5 μL Annexin V-FITC and 10 μL PI for 15 min at room temperature in darkness. Samples was analyzed by flow cytometry (BD FACScalibur Flow Cytometer, BD Biosciences) (Ex = 488 nm, Em = 530 nm).

#### 3.8.4. Caspase Activity Assay

Caspase-3, -8, and -9 activities were determined using the caspase-3/8/9 assay kit (Abcam) according to the manufacturer’s instructions. In brief, the A375 cells were exposed with inclusion complex or curcumin at different concentrations for 72 h. After reaching the specified time, the A375 cells were suspended in 50 μL of chilled cell lysis buffer and incubated cells on ice for 20 min. The mixtures were centrifuged at 10,000× *g* for 1 min, then the supernatants were collected and the total protein concentration was measured immediately by BCA kit. After that, cell lysates were added to the 96-well plates (every well containing an equal amount of protein) and 50 μL of 2×Reaction Buffer (containing 0.5 μL of 10 mM DTT) was added to each sample. Then, 5 μL of the Caspase-3 substrate (DEVD-p-NA), Caspase-8 substrate (IETD-p-NA), Caspase-9 substrate (LEHD-p-NA) were added, and incubated at 37 °C for 2 h. The samples were measured at 405 nm using a Multiskan MK3 microplate reader (Thermo Fisher Scientific, USA). The fluorescence intensity of the treated samples was compared with that of control samples to determine the fold-increase in caspase activity.

### 3.9. Statistical Analysis

The obtained data were expressed as the mean ± standard deviation of triplicate determinations. Statistical analysis was performed using the SPSS statistical package (SPSS 13.0 for Windows; SPSS, Inc., Chicago, IL, USA).

## 4. Conclusions

In this study, the results of XRD, FTIR, DSC, and UV showed that curcumin/cyclodextrin polymer inclusion complex had different physicochemical characteristics from free curcumin. After the formation of inclusion complex, the complex exhibited novel antioxidant and anti-cancer activities.

## Figures and Tables

**Table 1:** IC_50_ of different cells exposed to curcumin/β-cyclodextrin polymer inclusion complex for 72 h.

Cells	IC_50_ (μg/mL)
A375	476.4
A549	517.2
Hela	545.7
MCF-7	692.8
